# Confession to Make: Inadvertent Confessions and Admissions in United Kingdom and United States Police Contexts

**DOI:** 10.3389/fpsyg.2021.769659

**Published:** 2021-12-06

**Authors:** Luna Filipović

**Affiliations:** School of Politics, Philosophy, Language and Communication Studies, University of East Anglia, Norwich, United Kingdom

**Keywords:** inadvertent admissions, inadvertent confessions, inference, police interrogation, police interview

## Abstract

Previous studies have addressed many different kinds of confessions in police investigations – real, false, coerced, fabricated – and highlighted both psychological and social mechanisms that underlie them. Here, we focus on inadvertent confessions and admissions, which occur when a suspect appears to be confessing without being fully aware of doing so, or when police officers believe they have a confession or admission of guilt when in fact this is not the case. The goal of the study is to explain when, how and why these confessions and admissions occur as well as how they are dealt with in two different jurisdictions, the United States and the United Kingdom. We use a discourse analysis approach because inadvertent confessions and admissions of guilt are the product of miscommunication – they happen because the speaker’s meaning and the hearer’s meaning are misaligned. The data consist of 50 interviews from the United Kingdom and 50 interrogations from the United States with both English-speaking and non-English speaking suspects. Our results demonstrate that inadvertent confessions can occur in both locales due to reliance on inference, which is inevitable since inference is the backbone of any human communication, as well as due to additional factors such as linguistic, cultural and procedural issues. We found that these phenomena are more frequent and less well controlled for in the United States context due to (a) no systematic checking of understanding, (b) adversarial questioning techniques and an absence of legal representation, and (c) lack of professional, high-quality interpreting. We discuss the implications of our findings for current efforts to improve access to justice, custodial procedures and language services, and we make recommendations for the implementation of our research in professional practice.

## Introduction

How do you confess to something? The most obvious answer is by saying “I confess to X.” However, this happens rarely in police questioning of suspects. Confessions are elicited in a variety of ways and sometimes the persons confessing are not really aware that they are doing so, or what precisely they are confessing to. Furthermore, police officers may think during a communicative exchange that an admission of guilt has taken place, but upon subsequent examination of the conversation recordings or transcripts it turns out that there was no admission. Why does this happen? And more importantly, can it be prevented since the consequences of such occurrences for justice in general and for individual lives are potentially severe? This paper reveals the sources of, and the reasons behind, instances of misunderstandings, or lack of understanding, that lead to *inadvertent confessions* in both the United Kingdom and the United States policing contexts and illustrates how they can be prevented or dealt with properly after they have occurred.

Confessions in legal contexts have been studied extensively and from different perspectives. For example, research in the social sciences, most notably social and cognitive psychology, has helped us understand the circumstances that are more vs. less conducive to confessing, where the main factors that have been identified include strength of evidence, questioning style, rapport-building success (see e.g., Milne and Bull, 1999; Leahy-Harland and Bull, 2017). Previous literature has discussed coercion in confession elicitation and the specificities of police communication with suspects from both majority and minority linguistic and cultural groups (e.g., Kassin and McNall, 1991; Gudjonsson, 2003, 2006; Berk-Seligson, 2007, 2009, 2011, 2016; Filipović, 2007, 2019; Leo, 2008; Briggs and Russ, 2018). Many studies have also addressed the problem of false confessions (Gudjonsson, 1989; Kassin, 1998; Leo and Ofshe, 1998; Leo, 2001; Drizin and Leo, 2004). Who makes false confessions was shown to vary depending on many factors, such as personality, culture, mental state, sobriety and age. People sometimes do it deliberately to protect others. Eagerness to please or to conform in social situations is another factor, and so is mental illness or limited intellectual capability (Gudjonsson, 2003; Kassin and Gudjonsson, 2004). Another problematic type of confession involves fabricated confessions in which the alleged confessions of the perpetrators did not reflect their actual statements (either at all or to a considerable degree) but were rather based on the formulations made by the police officers who were questioning them (Eades, 1994, 2004, 2008; Coulthard, 2002).

The present research brings a somewhat novel perspective and a new insight into this interdisciplinary area. It does not focus on coerced or false confessions, or on the reasons why both innocent and guilty suspects may or may not confess, but rather on *inadvertent confessions* that happen when one party in a communicative exchange is not aware that a confession is being inferred and accepted as such based on what was said. In other words, we look at when, where and why *unresolved miscommunication in police communication with suspects* takes place and affects both interlocutors: on the one hand suspects are not aware fully (or even at all) that what they are saying is interpreted as a confession or as an admission of guilt, or about what precisely they are confessing or admitting to, and on the other hand, police officers may think that they have obtained a confession or admission of guilt when in fact this is not the case.

Another important novelty in this research is that it is not based on a single case study or single jurisdiction, as is most often the case in linguistic analyses of police interviews or police interrogations. The present study involves a substantial database from both United Kingdom and United States jurisdictions, namely 50 transcripts of United Kingdom police interviews and 50 United States police interrogations. In addition, the data set includes both monolingual and bilingual (interpreter-assisted) cases, which is very rare in the field. In this way, we will be able to see *how often* a certain type of miscommunication can be interpreted as an admission of guilt or as a confession, and which aspects of language structure and use can become *sources* of miscommunication.

Inadvertent confessions can be coerced but need not be, as we shall see in this paper. They can sometimes occur as the outcome of an unbalanced power play, or of emotional pressure during questioning, but they can also arise due to insufficient proficiency in the language of the interview or due to difficulties in providing an exact translation. Whenever suspects are apparently not aware that they are confessing to something, or when what they are saying will be interpreted as a confession, or when they are not fully aware of all the details of the confession, we can say that such confessions or admissions are *inadvertent*.

We must point out here that in this study we make a *distinction between confession proper and admission of guilt* (Gudjonsson, 2006) but include both in the analysis because confessing to an offense does not always have the format of a full confession, as we already indicated. Gudjonsson (2006) highlights the problem of confessions being defined differently in different studies, which makes cross-comparison difficult. This is mainly due to the fact that a precise distinction between “admissions of guilt” and “confessions” tends not to be drawn. If defined very broadly (Drizin and Leo, 2004: 892) “any statements which tend to incriminate a suspect or a defendant in a crime” could be considered a confession. This definition also includes denials, which, as Gudjonsson (2003) explains, causes great confusion in the field. Gudjonsson (2006) suggested a narrower definition, which he argues is also more correct from a legal point of view: a “confession” would be defined as “a statement admitting or acknowledging all facts necessary for conviction of a crime” and an “admission” as “an acknowledgment of a fact or facts tending to prove guilt which falls short of an acknowledgment of all essential elements of the crime” (ibid.). This distinction would exclude self-incriminating admissions from the confessions pool because they do not amount to explicit acceptance of full responsibility by the suspect.

We agree in principle with this distinction between a confession and an admission of guilt and the rationale for it. However, for our present analysis, we will include both confessions and admissions of guilt as our primary focus. Both confessions and admissions can happen inadvertently when given by the speaker or when inadvertently interpreted as such by the hearer, and crucially, they can *significantly impact* further developments in police questioning as well as potential case outcomes. The phenomenon of inadvertent confession and admission of guilt has been studied before to some extent, for example, in the context of self-implication due to manipulative questioning (see Berk-Seligson, 2009; Filipović, 2019, 2021), inadequate language support for suspects from minority language backgrounds (Eades, 1994, 2008, 2012; Berk-Seligson, 2007, 2009, 2016; Filipović, 2007, 2013, 2019, 2021) or as a result of patient and meticulous sequential introduction of incriminating evidence in United Kingdom police interviews (Filipović, 2019; Musolff, 2019). What the present study brings is *a holistic and comparative view of two very different approaches to police questioning* as well as *contrastive insight into monolingual vs. bilingual police communication* and the *effect of all these different factors on multiple inadvertent confessions and admissions*. As a result, we will be able to see, in both qualitatively and quantitatively informative ways, what the reasons are for these disturbing occurrences that jeopardize the pursuit of justice and we will be able to suggest ways for preventing and remedying them.

Our analysis is contextualized within a linguistic pragmatic theory of meaning, whereby conveying meaning includes both what is inferred as well as what is explicitly stated (Grice, 1957, 1975, 1989). Grice’s pioneering work and the extensive subsequent pragmatic research that has built on it have demonstrated that interlocutors habitually rely on inferences and that this reliance on inferences is typical for all aspects of human communication, across different languages, cultures and social contexts. Inferences are based on implied information, which makes them malleable to different interpretations and subject to easy denial. Most of the early research in pragmatics argued that the meaning conveyed in conversation consists of retrieving the intentions of the speaker, i.e., making the correct inferences about what the speaker wanted the hearer to understand. However, more recent research has brought the hearer to the fore and argued that it is equally legitimate to assume that the meaning that is conveyed is indeed what the hearer inferred, which is not always in line with what the speaker intended. Thus, meaning is seen as the product of *negotiation* (see e.g., Elder and Haugh, 2018), which, like any negotiation, may or may not result in agreement. We shall see that the consequence of apparent (but not explicit or clear) agreement on what was meant, and possible disparities between interlocutors regarding what was (potentially or actually) inferred, creates miscommunication that can cause serious adverse outcomes for those involved as well as for the criminal justice system as a whole.

## Previous Relevant Literature: a Selective Overview

### Interrogation vs. Interviewing of Suspects

If we want to draw the most basic distinction between United States and United Kingdom techniques for police questioning of suspects, we can say that United States police interrogate their suspects while United Kingdom police interview them, though in practice this divide is not always so clear-cut. In the United States context “interrogation” is principally used in the literature and in police practice to refer to the questioning of criminal suspects, whereas the term “interviewing” is more commonly used for witnesses and victims. Williamson (1993) proposed the term “investigative interviewing” to cover the interviewing of both witnesses and suspects. This term is now used to cover all types of interviews in the United Kingdom, which reflects a shifted focus on gathering reliable evidence rather on obtaining a confession, which is still the primary focus of the interrogation-based method practiced in the United States (see also Williamson, 2006). Namely, interrogations in the United States are characterized by an adversarial approach based on the Reid Technique^1^ and are primarily aimed at eliciting confessions. It is a highly confrontational and accusatory process of interrogation and consists of a nine-step approach (Kassin and Gudjonsson, 2004; see Gudjonsson, 2006 for a clear and succinct description and critical discussion). Interrogation is also a process that presumes guilt and whose goals are to fight against denials, break down resistance, and increase the suspect’s desire to confess (e.g., Inbau et al., 2001), whereby different persuasion strategies are used in order to convince suspects that it is in their best interest to make a confession (Leo, 1996). In order to achieve this objective United States police may engage in deception, such as exaggerating the seriousness of the offense or the strength of the evidence against the suspect, and present suspects with false evidence that apparently incriminates them, etc.; see Kassin, 2006). Such activities are illegal in the context of United Kingdom policing. In contrast, the United Kingdom approach to interviewing suspects has its foundation in the PEACE approach^2^, which outlaws oppressive techniques by favoring open questions and bans the use of deception strategies such as presentation of false evidence. Its aim is to obtain the highest quality testimony for investigative and evidentiary purposes rather than confessions without corroboration (Milne and Bull, 1999). There is some evidence supporting the validity of the United Kingdom approach to police interviewing (Clarke and Milne, 2001) though its full implementation in practice may not always be easy or successful (Griffiths and Milne, 2006; Dando et al., 2009). In terms of cases of confessions, Meissner et al. (2012) show in their overview of published studies that false confessions are more frequent in the United States than the United Kingdom and the communication method is likely to have something to do with this finding. Gudjonsson (1992) used extensive research-based evidence and numerous case studies to highlight the risk of false confessions occurring during custodial interrogation. He raised further concerns, such as ethical issues that arise with the Reid Technique, including the use of trickery, deceit and dishonesty as a way of breaking down resistance (see Gudjonsson, 2003). A recent survey of experts on the psychology of confession by Kassin et al. (2018) revealed that the risk of false confessions increases not only because of explicit threats and promises but also when two common tactics are used, namely the introduction of false evidence and minimization of the severity of an outcome in the form of an offer of sympathy and moral justification accompanied by an implication of leniency. Such tactics are not allowed in the context of United Kingdom policing but Pearse and Gudjonsson (1999) noted that even in the British contexts, in the more serious cases, the dynamics can change considerably from the prescribed interview approach and Reid-like approaches are used instead to break down resistance, often rendering a confession inadmissible at trial. This chimes with some earlier observations by different authors. For example, Stephenson and Moston (1994)^3^ found out that most of the United Kingdom investigative interviewers who were surveyed (80%) still believed that the main purpose of an interview with suspects was to elicit confession. Things have moved on since then and improvements have been made through PEACE-focused training (Clarke and Milne, 2001), but it has to be pointed out here that obtaining good-quality information, as opposed to obtaining a confession, remains particularly challenging for the interviewer (Oxburgh et al., 2016). Namely, he or she must make allowance for the substantial institutional power held over the interviewee and create an environment that is conducive to the elicitation of evidence unimpeded by the fact that this is done by a representative of that very power – the interviewer (see also Vanderhallen and Vervaeke, 2014). As Oxburgh et al. (2016) explain, the interviewer is the dominant participant in the interview, even when he or she does not intend to be, because of the nature of this specific interactional context which actively reinforces this dominance.

It is also important to note that persuasive tactics by the police may not be the only or even the main reason why guilty suspects confess. Some earlier studies have found that most suspects who wanted to confess did so in the beginning of the interview (Baldwin, 1993), and a “sizable proportion” of those who are impacted by the interviewing tactics may likely be false confessors (Milne and Bull, 1999). Some key factors for confession have been identified, namely strength of the available evidence, access to legal advice and previous criminal history, and severity of the offense (Sweeney, 2017:164). However, a study in Canada found that 25 percent of suspects changed their mind about confessing during the interview, though the changes did not go only in the direction of a confession: half of these suspects eventually decided not to confess after initially wanting to do so (St-Yves and Deslauriers-Varin, 2009: 2). By the same token, prisoners surveyed by Gudjonsson and Petursson (1991) stated that external pressure coming from the police interviewing style was one of the main factors in their confessions, while others included internal pressure brought on by a sense of guilt and by the strength of the evidence against them. In a recent study Bull and Baker (2020) found that what works best in obtaining a confession in the United Kingdom policing context is non-coercion and the communication of respect.

In any case, interview techniques are important, as Sweeney (2017: 165) points out, if for no other reason than to prevent a person who is willing to confess from changing their mind. In addition, interview techniques are important for one other reason: they can make a difference between a suspect shutting down vs. opening up (Musolff, 2019), and it is always better to keep the communication channel open. Even suspects who are not willing to confess can provide invaluable additional evidence (ibid.).

Interestingly, in spite of the greater restrictions imposed on police interviews in England, the overall confession rate has not been reduced and it remains considerably higher than the confession rates reported in the United States (Gudjonsson, 2003; Kassin and Gudjonsson, 2004). The evidence suggests that suspects confess for three main reasons – awareness of the strength of the evidence against them, internal pressure, and custodial and interrogative pressure (including deceit, trickery and psychological manipulation). Awareness of the strength of evidence is the single most important reason. This has important implications for investigators. Where the evidence against the suspect is weak or flawed, interrogative and custodial pressures increase the risk of false confessions. Williamson (2006) warns against overreliance on confessional evidence in both adversarial and inquisitive jurisdictions and warns that confession-focused questioning may lead to miscarriages of justice.

### Vulnerability and Disadvantage in Police Communication With Suspects

In the context of this study it is important to consider the specific issues that pertain to interviewing vulnerable suspects because these suspects are more at risk of incriminating themselves without being aware that they are doing so (see Gudjonsson et al., 1993). We actually know that investigative interviewers are not provided with guidance on how to carry out interviews with vulnerable suspects (O’Mahony et al., 2016). This is particularly problematic because previous research has shown that communicative challenges for vulnerable interlocutors will likely result in a great deal of confusion, even for someone without a cognitive disability (Harres, 1998; Blankenship and Craig, 2007), and more so for children and adults with an intellectual disability. In particular, some frequently used formulations, such as coercive tag questions (as in “you hit her, didn’t you?”) can have the further negative effect of forcing the interviewee to agree with the interviewer (Harres, 1998). Acquiescence by a vulnerable person and inclination to answer a question in the affirmative is often driven by the belief that this is the response wanted by the person in authority. Another reason why vulnerable suspects comply with a request is so that they can leave the police station as soon as possible (Gudjonsson, 2003).

Another documented source of vulnerability and disadvantage before the law is that of not speaking the majority language in which the conversation is taking place (Hales and Filipović, 2016). Previous literature has shown that speakers from a minority linguistic or cultural groups have more difficulties in legal procedures than the majority language speakers or majority culture members. For instance, Fowler et al. (2016) cite “the language barrier” as a commonly identified negative factor in police interviews. Police officers tend to see communicating with a person who does not speak the majority language as much more problematic than communication with native speakers (ibid.). A further difficulty specific to the context of United States policing is the fact that bilingual police officers often act as interpreters in suspect interviews, which means that these suspects are not receiving the unbiased language support that they need and that they should be getting (see sections “Results and Discussion” for examples and further details).

Past research has reported multiple instances of communication problems in both monolingual and bilingual, interpreter-mediated police interviews and interrogations (Filipović and Hijazo-Gascón, 2018; Filipović, 2019; Musolff, 2019), though bilingual communication contains more numerous challenges. Experimental research in the United Kingdom context has shown that monolingual interviews in L2 (second-language) English provide more information relevant for the investigation than interviews done in L1 (mother tongue) via an interpreter (Grzybek, 2017). However, it is fundamental to establish prior to any official communication that the speakers do indeed have enough L2 proficiency and understand all the details of what is communicated in a sensitive context such as a criminal investigation. It is not enough to have basic interactional proficiency in the L2 as many scholars have pointed out (e.g., Berk-Seligson, 2002; Eades, 2008, 2012, 2018; Filipović and Abad Vergara, 2018, among others). As previous research has also shown, even fluent monolingual speakers who speak the language of the justice system in which they are processed (e.g., English) have difficulty understanding some key legal concepts such as the Miranda Rights in the United States or Caution in the United Kingdom (Pavlenko, 2017; Berk-Seligson, 2016; see also example (3) in the next section and the “Conclusion and Limitations” section).

A number of studies on United States police data have shown that there are serious problems for justice when native speakers of Latin American Spanish are interviewed either in their first language, Spanish, by United States Spanish speaking police officers, or in their second language, English (Filipović, 2007; Berk-Seligson, 2011; Filipović and Abad Vergara, 2018; Hijazo-Gascón, 2019; Dumas, 2020). Many serious (and potentially fatal) instances of miscommunication occur in such interrogations due to officers’ insufficient proficiency in the relevant other language, or to inadequate translation, or poor understanding of English as a second language if English is used. It is not only a minority language but also a minority culture that can be a source of problems for people who are being questioned in legal contexts. Many minority cultures, such as Australian Aboriginal (Liberman, 1981; Eades, 1994, 2008; Cooke, 1995) and Meso-American social groups (Ross and Mirowsky, 1984; Solan and Tiersma, 2005; Berk-Seligson, 2009), especially those of lower socio-economic status, use *gratuitous concurrence* as a protective device, even when things are not being understood well or even at all. This behavior is in line with culturally driven strong aversion to open conflict and contradiction to authority within a number of minority communities. Eades (1994, 2004, 2008, 2012) reports numerous cases from Australia where the alleged confessions by Aboriginal English speakers were discovered to have been fabricated by the police because they contained statements that could not have been phrased as such in Aboriginal English and alleged references that in fact were culturally unwarranted, such as citations of precise time on the clock (which is not done in the Aboriginal culture in question). Overall, linguistic and cultural minorities do seem to have an additional disadvantage in legal systems around the world, which academic research has identified and helped raise awareness about, and this can then lead to improved outcomes and rectifications of a number of miscarriages of justice (see Eades, 1994, 2012; Coulthard, 2002; Berk-Seligson, 2007, 2009).

## Theory, Data and Methodology

### Theoretical Background

In this study we focus on the level of meaning in communication, which falls in the interdisciplinary research domain of *language, the mind and the law*, with a focus on how interlocutors create meanings in interaction and the effects and consequences of these interactions in different social contexts, in this case the context of law enforcement (Filipović, 2019; de Pablos-Ortega, 2019; Pounds, 2019). One of the key insights in the study of human communication has been that context allows hearers to derive an appropriate interpretation of what the speaker has said since words and sentences can mean different things in different contexts – they are underspecified or ambiguous, and context needs to be relied on in order to select the intended sense. Context can also lead to particular inferences being drawn, which were termed *implicatures* by Grice, 1957, 1975, 1989, and these form the basis for deriving the pragmatic meaning of an utterance. According to Grice, understanding the meaning of an utterance consists of retrieving the speaker’s intentions behind it. Here, we adopt a somewhat different definition of meaning and consider it to be an output of a negotiation between the interlocutors in concrete conversational situations (Elder and Haugh, 2018), whereby multiple interlocutor turns may be needed in order to determine if agreement (i.e., shared understanding) has been achieved in the negotiation. This is because conveying meaning consists of something more than just retrieving speakers’ intentions – it is also the hearers’ subsequent responses and their intentions that count, which may or may not align with the speakers’, and this is when miscommunication takes place. Interlocutors can have misaligned communication goals due to different personal or institutional motivations, for example revealing or discovering the truth (police officers) vs. obscuring the truth (suspects). This then results in ignoring and disregarding unwanted yet possible or obvious interpretations of what is said (accidentally or on purpose) or drawing inferences from the hearers’ responses that may have negative consequences for them but serve the purpose of the speakers (again, accidentally or on purpose). It is possible that either one or both sides fail to detect that a miscommunication has taken place because they are firmly attached to their preferred interpretations and not aware of the different understanding of their interlocutors. It is also possible that one or both interlocutors detect the miscommunication but decide not to engage in further negotiation of meaning and instead let the miscommunication persist (see Filipović, 2021). We will not focus here on determining whether miscommunication on the part of either police officers or suspects was purposeful or manipulative (but for further detail see Kassin and McNall, 1991; Filipović, 2007, 2013, 2019; Musolff, 2019). Here, we focus on the precise points in exchanges in which inadvertent confessions and admission of guilt appear to take place and on the real or possible consequences for case outcomes. In addition to our focus on the role of *inferencing*, we look at other factors that may lead to inadvertent confessions and admissions, such as *translation issues* in bilingual communication, and *procedural issues*, such as the availability of professional language services and employment of the accusatory vs. information-gathering approach in police questioning.

### Data and Methodology

The database used in the current study consists of 50 United States police interrogations and 50 United Kingdom police interviews with suspects who are monolingual speakers of either English (United Kingdom data) or another language (Spanish in the United States portion of the dataset and Russian, Lithuanian, Polish or Portuguese in the United Kingdom data subset). The sample collection was random, based on what each jurisdiction decided to make available. Two United Kingdom constabularies and three United States districts from the state of California contributed to the database, which includes only closed cases from 2006 to 2016. The average length of the transcribed data is 27 pages per document. The content of each transcript is also variable, from only “no comment” answers throughout the interview to rich, long narratives and detailed responses to questions. The United States transcripts were all produced verbatim (i.e., every verbal and non-verbal signal was recorded in writing) and in the case of interpreter-assisted interrogations the transcripts were produced bilingually in Spanish and English. All the United Kingdom transcripts are only monolingual in English only and non-verbatim (with sometimes as much as 20 min of conversation missing), and because of this it was essential to refer both to the original tape recordings of the interviews and to the transcribed data when analyzing the United Kingdom portion of the database. The United States recordings were available in only 9 out of 50 cases and were only randomly checked to ensure that verbatim transcription was adhered to throughout, which was indeed the case. The topics discussed in the United Kingdom and the United States data overlap to a great extent, though overall, the United States cases contain a higher number of exchanges in the context of more serious crimes (murder, incestuous sexual assault, rape, assault with a deadly weapon) while in the United Kingdom the offenses involved are more mixed and include both serious (e.g., possession of child pornography, sexual assault, domestic violence and robbery) and less serious ones (e.g., supermarket theft and car insurance fraud).

The materials were studied in the following way. All the transcripts were read in detail by the lead researcher, and only by her, since permission to access the data was granted based on individual and detailed security checks. A partial inter-rater reliability check was nonetheless performed on a portion of the dataset (20 out of 100 files), to which another researcher had access^4^ and the reliability rate on that portion was 100%. For United Kingdom data the CD or memory stick recordings were played as well to ensure that data points of interest were not missed in the non-verbatim transcript. *Inadvertent confessions* were detected when suspects were expressing agreement or concurrence with a police officer *without themselves explicitly stating or demonstrating awareness of* what exactly was being confessed to. *Inadvertent admissions* of guilt occurred when the admission of guilt was not clearly and explicitly given by the suspect but was rather *inferred and acted on as such* by the police. The analysis given here is primarily qualitative though some relevant quantitative observations will also be made. All of the detected instances are exemplified, discussed and illustrated in the next section.

## Results and Discussion

We found a total of 17 confessions and admissions of various types, 11 of which contained the specific types that we are focusing on here, namely inadvertent confessions or inadvertent admissions. Out of these 11 cases, 5 were full confessions (4 in the United States and 1 in the United Kingdom data) while the rest were admissions of guilt for parts of the alleged crime (3 in each data portion, the United States and the United Kingdom). The rest of the confession and admission cases (6) were not of interest to us at present because they contained confessions or admissions that were given with full and explicit consent of the suspect.

The immediately noticeable finding is that inadvertent confessions and admissions constitute the majority type in dataset, 11 out 17. Furthermore, it is also noticeable that the overall number of inadvertent confession cases in the United States data is significantly higher than in the United Kingdom data (4 vs. 1). A higher number of cases in the United States data is not unexpected since the previous literature has reported that some confession types, e.g., false confessions, are much more frequent in United States policing contexts (Meissner et al., 2012). Our own earlier empirical research (Filipović, 2019, 2021) has shown that instances of miscommunication are overall more frequent and more frequently exploited in United States suspect interrogations than in United Kingdom suspect interviews. We have a further indication here that the approach to questioning (United States interrogation vs. United Kingdom interviewing) does seem to play a role in the current study. It may not be the crucial factor for the overall number of confessions in general in the United States vs. the United Kingdom since, according to Gudjonsson (2006), the overall number does not significantly differ in the two policing contexts, but it appears to matter when it comes to the *kind of confession* that is being elicited. The present analysis reveals that the same shared communication phenomena are at play in both jurisdictions with regard to confessing or admitting guilt inadvertently, though we also detected very important differences with respect not only to the number of cases but also to the ways in which the issues are dealt with, which we discuss in detail in the next two sub-sections (*Inadvertent confessions* and *Inadvertent admissions of guilt*).

### Inadvertent Confessions

We first focus on the five cases of apparently full confessions, which we consider to be inadvertent because the suspects were either not aware that their communication was treated as a confession or were not fully aware of what exactly they were confessing to.

The first case we look at here is an apparent confession by a suspect accused of a very serious crime (repeated sexual assault of a minor) and the victim involved was a family member (the suspect’s daughter). The interview takes place in Spanish, with one of the police officers acting as an interpreter for the benefit of another police officer who is mainly involved in doing most of the questioning in English. The police officer-interpreter also asks the suspect some questions independently in Spanish and repeatedly fails to render the whole content of the suspect’s answers into English. This dual role of the officer-interpreter is a problematic issue in its own right and previous studies have pointed out some real-life consequences and potentially severely damaging outcomes for suspects when such a dual role is assumed by police officers in the United States (e.g., Berk-Seligson, 2009, 2011, 2016; Filipović and Abad Vergara, 2018; Filipović, 2021). It is relevant to mention here that this kind of inherently biased and unqualified language provision for speakers with *zero or limited English proficiency* (i.e., ZEP/LEP speakers) is not permitted in the United Kingdom context of police interviews, where only registered and certified professional interpreters are used.

Throughout this lengthy exchange the suspect is clearly upset: there are multiple references in the transcript to the suspect crying, as illustrated by the example in (1) below. By the end of the interview he apparently confesses to every offense that the police officer has listed and described throughout the interrogation, which was allegedly based on an earlier account by the victim. Importantly, throughout this interrogation it was the police officer who was both describing the multiple offenses and offering a probable motivation for them (“you are not a monster, you did it because you loved her”), while the suspect was only expressing his emotions (e.g., “I was angry, lost control”). The suspect also kept asking the officers to stop (by saying “please no more” 9 times). The interview ends with the two police officers agreeing that they had a confession, but it is obvious from the example that the suspect is not fully aware of all the detail in the confession itself^5^ :

(1)
PO1: Is that all true?
PO2: Todo eso es la verdad?
[Everything is true?]
S: Si…*[CRYING]*
[Yes…]
PO2: Yes.
PO1: Is everything she’s telling me the truth?
PO2: Todo, todo lo que dijo ella es verdad?
[Everything, everything that she said is true?]
S: Yo no sé, tal vez ella dijo más, yo no sé. *[CRYING]*
[I don’t know maybe she said more, I don’t know.]
PO2: I don’t know if it’s the truth, I just answered what you wanted me to.
PO1: Ok, so you answered all the questions…

The miscommunication in the example (1) stems from the fact that the suspect’s and officers’ goals are clearly misaligned, and the interlocutors clearly have different intentions and neither side seems to acknowledge the communication needs of the other. The suspect appears to admit to everything in order to end this ordeal and because his pleas to stop the questioning are not being acknowledged, while the police officers are seeking, accepting and also accounting for the reasons behind the listed crimes throughout the interrogation.

The second United States case of inadvertent confession is similar in terms of the end result (confessing without realizing what exactly is being confessed to) but the process that led to it was quite different from what we saw in example (1). The offense is again that of a sexual assault of a minor though it does not include full sexual intercourse as in the previous case. This interrogation is monolingual, in Spanish only, and it consists mainly of an extremely lengthy monologue by the Spanish-speaking police officer, who speaks 98% of the time (in the longest transcript in the dataset of 100 pages). The suspect mainly just concurs throughout while the police officer uses every trick in the manipulation book: fraternization, minimization of the offense, maximization of adversarial outcome if confession not given presently (see section “Previous Relevant Literature: A Selective Overview”). Again, we observe specific patterns of interaction and the interpretation of back-channeling responses such as “um-hum” as agreement, as noted in the previous literature as well (e.g., Berk-Seligson, 2009). As we discussed earlier (see section “Vulnerability and Disadvantage in Police Communication With Suspects”) this response type is characteristic of some cultural groups and of disadvantaged ethnic minorities, and it is also common in police questioning due to the inherent inequality in power relations between the interlocutors (i.e., police officers and suspects). In the end the suspect hesitates when asked to confirm the confession as described by the police officer but the officer reminds him that he has already confessed anyway (by responding “um-hum”) and that it is in his best interest to confess. This is another case of misunderstanding the intentions of the interlocutor, whereby the suspect’s concurrence means agreement for the police officer but not for the suspect. In addition, the suspect has apparently misunderstood the police officer’s intentions and appears to be unaware of having made a confession during the interrogation. An excerpt is given in example (2)^6^ :

(2)
PO: Okay. Tu dimme en tus palabras mano. En tus palabras porque lo que quiero hacer, me entiendes, es de que tu me dijistes tu version, okay. Ah tu version, este quisiera que fuera 80 porciento por lo menos correcto.
[Okay. You tell me in your words bro. In your words because what I want to do is, do you understand me, is that you tell me your version, okay? Ah your version, I would like it to be 80 percent correct, at least.]
S: Um hum.
PO: Okay, esto no, no es el crimen del año, como te digo.
[Okay, this is not, not the crime of the year, like I tell you.]
S: Um hum.
PO: Tu crees que no conoszco mi raza? Yo era asi. Era bien volado.
[Do you think I do not know my race? I was like that. I was very wild.]
S: Um hum.
PO: Te ha provocado y tu tenias que reagir, verdad?
[She provoked you and you had to react, right?]
S: Um hum.

In the next case, also from our United States data subset, we have an inadvertent confession when the suspect answers “yes” to the officer’s question about the reasons for confessing to repeated physical assault of the victim (inappropriate touching including sexual assault). However, when the interrogation suddenly switches from English (the second, weaker language of the suspect) to Spanish (the suspect’s first and stronger language) we see that the suspect has not understood much of what has been going on in this interrogation up to that point and that the suspect’s earlier “confession” of having touched the victim inappropriately (as reported in the PO’s 2nd turn below and which is said to have been given because of the suspect’s DNA found on the victim) was probably given inadvertently, through miscommunication. This is likely to have happened due to the language barrier created by the complexity of the PO’s utterance (example 3, police officer (PO) second turn) and by the suspect’s limited command of English (evident in his ungrammatical speaking turns below, such as “the girl not say that”, the suspect’s third turn in example 3):

(3)
PO: And last time they told you that, the DNA was found on her, right?
S: Yeah.
PO: And it’s now; the DNA…that was found on her; was that the reason why you thought about it and said you touched her?
S: Yes.
PO: Yeah, you know, you can’t clean it up; not the DNA, okay? We are not yelling, we are not going to arrest you. We just want to know the truth. Was she pushy? Was she, you know, touch me here?
S: No the girl not say that. I am not talking nothing; no talking nothing.
PO: Well we don’t understand is why would your DNA be inside her vagina or her butt? Why?
S: I don’t know.
PO: Como, explique se? Como se podia explicar?
[How, explain yourself? How can it be explained?]
S: No sé, no sé.
[I don’t know, I don’t know.]
PO: No explica?
[You can’t explain?]
S: No sé, no entendido nada de esto tampoco.
[I don’t know, I didn’t understand none of this either].

In the next example, which is the last confession case from our United States portion of the dataset, we see another instance of a language barrier, which clearly demonstrates how LEP/ZEP suspects can be put in a more institutionally disadvantaged position than English-speaking suspects – they have even less power over what is communicated than suspects who communicate in the majority language, English (see Filipović, 2007, 2019, 2021; Berk-Seligson, 2011, 2016; Dumas, 2020)^7^ :

(4)
PO: Okay, and then what did you do with her?
INT: Y que pasó?
[And what happened?]
S: … se me cayó en las gradas.
INT: [… I dropped her on the steps.]
PO: Where did you drop her?
INT: Donde la botaste?
[Where did you throw her?]
S: Aqui…
[Here…]

In this example (4) the suspect was clearly stating that the dropping of the victim was unintentional because this is the meaning of the Spanish *se* + *dative constructions*, as in the suspect’s “se me cayó,” literally “it happened to me that she fell” (see also Filipović, 2007, 2013; Ibarretxe-Antuñano, 2012). There is no adequate equivalent for this construction in English and the verb “drop” used in the translation is actually a legitimate translation choice. The problem is that the verb “drop” in English is unspecified and vague with respect to intentionality – it can refer to either an intentional act of dropping or to an unintentional one and can be understood either way in this case. It is important to emphasize here that the suspect was not denying his involvement in this incident, but he was clearly stating that *he did not do anything on purpose*. However, when the police officer uses the verb “drop” for the second time in his question “where did you drop her?” the interpreter uses an intentional verb in Spanish, “botaste” which means “threw on purpose” and the suspect shows the location, and through this translation instance the verb “drop” becomes linked to its intentional interpretation. The suspect is thereby inadvertently confessing to a much more serious crime, that of throwing the victim down the stairs, which resulted in the victim’s death.

Our last example of an inadvertent confession is the only one from the United Kingdom data and it is actually an instance of an apparently inadvertent confession that was challenged by the investigating officer. This case from the United Kingdom is unlike the previous cases from the United States because the United Kingdom police officer *insists on re-visiting the apparent confession* in order to ascertain that it was actually meant by the suspect. This is very important to highlight because in the United States the interrogation normally stops once the confession has been obtained. In the United Kingdom the interview does not stop there but continues in order to ensure that the confession or admission of guilt was given with full awareness and can be supported by evidence. This is illustrated in the following example, where the offense involved is a theft (of alcoholic beverages from a supermarket):

(5)
PO: With regards to the statement “I have no money, I was going to sell it,” what did you mean by that?
S: That I didn’t have any money on me and that I was going to sell them at a profit.
LR: Normally he would buy it and sell it but he had no money and obviously his English is not brilliant^8^.

It is interesting to point out here that the suspect seems to confirm his confession and the intention to offend but his legal representative steps in to ensure that this “confession” should be reconsidered due to his poor command of English. The legal representative also seems to be arguing that there was *no intention* to steal by explaining further in the interview that the suspect would normally buy and re-sell, but on this occasion he realized too late that he had no money once he filled his cart with the goods. This interaction means that the earlier confession by the suspect, that he intended to steal and re-sell, was due to a miscommunication and has now been put in doubt. We can conclude here that the communication style of the police officer (which involved checking to ensure that an earlier inference drawn was correct) as well as the presence of legal representation resulted in a confession being averted.

### Inadvertent Admissions of Guilt

We now turn to examples that are not confessions according to our narrow definition (see section “Introduction”) but rather *inadvertent admissions of guilt* about *one specific detail of the alleged crime*. In the following examples (6 and 7) from our United Kingdom data the exchanges were about whether the suspect knowingly committed an offense, namely possession of a knife in example (6) and downloading illegal sexually explicit material in (7). In the former case the apparent admission was given by the suspect, we would argue, inadvertently, without knowing what was being admitted precisely. The suspect had been accused of domestic violence, and possession of a weapon (knife) was a possible additional offense. He keeps concurring with the investigating officer, and as we discussed earlier (see section “Vulnerability and Disadvantage in Police Communication With Suspects”), concurrence is often interpreted as agreement in this kind of situation. Fortunately, such an interpretation was not the case here. A potential issue with the suspect’s full understanding and awareness about the definition of the alleged offense was immediately noted by the police officer, and also questioned by this suspect’s legal representative, so the inadvertent admission seems to be revoked:

(6)
PO: It is an offense in this country to be in possession of a knife in a public place. Do you understand that?
S: Mmm.
PO: Were you aware of that?
S: Mmm.
PO: I suppose not.
LR: It is not a blanket offense is it?
PO: No but I mean it is different from the story you have given me to the story what the witnesses are saying because the witnesses are saying that at one stage as the paramedic shout out you have got a knife you are seen trying to stuff it down your trousers.

In the next case (7) the legal representative also intervenes, preventing an admission at first, but when the officer returns to the topic the suspect inadvertently confirms that he was indeed aware of the illegal content being downloaded:

(7)
PO: I am not asking you how many, I am asking you is there content on the computer that involves sexual activity between two people?
S: Probably.
PO: Would that involve a person who you would reasonably believe to be under the age of about 16?
LR: In the light of what we discussed you don’t have to answer that question.
S: I’d rather not answer that.
*[7.4 min later]*
PO: What nature was it [the downloaded material]?
S: Childporn.

In the case of (6) it is obvious that the police officer was willing to give the suspect the benefit of the doubt, namely that the suspect may not have knowingly committed an offense. So even though the suspect apparently admitted to knowingly committing the offense in question, the police officer assumed that the suspect may not have been fully aware of the exact contents of the offense that he apparently admitted to. Unlike the example (2) from the United States, the police officer does not take concurrence to mean understanding or agreement and instead assumes that the suspect is not able to draw the relevant inference and understand the meaning that the police officer was trying to convey (i.e., elicitation of a confirmation that an offense was knowingly committed). In addition, a further clarification (that carrying a knife is not a blanket offense *per se*) is also appropriately sought by the legal representative. In contrast to this example, the initially averted admission of guilt in (7) is subsequently given apparently inadvertently when the police officer revisits the same topic. The suspect in this case was trying (and being advised by the legal representative) throughout the interview not to reveal whether he was aware of the fact that the content on the images and videos he had downloaded involved children. Then later, when the legal representative did not intervene, the suspect answered (the last question in the excerpt 7) in a way that attests to his ability to make a judgment about whether children were involved. This suspect clearly had no intention of admitting guilt because he continued to mention that he was not able to make a distinction between a 10 and a 16 year-old child. However, the use of the word “childporn” clearly signals that the suspect was very much aware of the fact that he believed children to have been featured in the video materials in question. Thus, we can say that in this case the inadvertent admission happened in spite of the intention not to admit.

It is important that police officers take note of cases such as (6) and (7), because in the former case (6) the admission may not have been warranted, as it appears to be based on the lack of a proper understanding, but in the latter case (7) the apparently inadvertent admission is very revealing as it undermines the suspect’s claims about not knowing who can be considered a child. Case (7) also illustrates how keeping the communication channel open and revisiting a subject with a reluctant interlocutor may result in the elicitation of information that is of key investigative value (Musolff, 2019). The two examples are also similar to the extent that in both cases the respective police officers can potentially draw negative inferences about the suspects’ committing the crimes knowingly, and both suspects failed to prevent, detect or act on these possibly damaging inferences.

The following two examples (8 and 9) also illustrate the inadvertent admission of guilt, but they differ from the previous two cases (6 and 7). In (6 and 7) the admission of guilt is apparently verbally given but perhaps without full awareness of the significance of the relevant responses (7) or without full awareness about the definition of the offense and its legal implications (6). In examples (8) and (9) the admission of guilt is not explicitly given but appears to be created via an inference made by the police officer who is interviewing the suspect. The statements by the suspects in both cases (8 and 9) seem to be treated as an assumption of guilt by the police officers, because the questioning continues as if the admissions that are sought have already been given. However, neither of the suspects does actually provide the relevant admissions – they were inferred by the police officers, and as we know from pragmatic theory in linguistics, inferences are easily cancelable (Grice, 1989). In other words, the reason behind these *apparent* inadvertent admissions is the general principle of reliance on inferences in any conversational exchange, including police contexts. As discussed in the “Theoretical Background” section, meanings are created in negotiation between the two interlocutors and are driven by their respective inferences that may or may not be matched. It is because more than one inference is possible in many situations, and because the different inferences drawn in the same situation can be mismatched that we cannot hold others accountable if inferences different from ours are made. Crucially, our interlocutor can easily cancel the inference we derived by offering an equally acceptable alternative (see Filipović, 2021, for details). The police officers in these two cases apparently treat such instances as admissions and build on them further in subsequent parts of the interrogation (8) or interview (9), while the actual unequivocal admission of guilt remains unelicited:

(8)
PO: Yo sé que tu eres Sureño y no es un gran secreto para mi.
[I know that you’re a Sureño and it’s no big secret to me.]
S: Yeah.
[Yeah.]
PO: …me entiende…
[…you know what I mean…]
S: …nada, pues…
[…nothing, you know…]
PO: Yo sé que no es un gran secreto.
[I know it’s not a big secret.]
S: Pues, es tu trabajo.
[Well, it’s your job.]
PO: Donde se juntásteis antes de montar el coche para salir ….
[Where did you (plural) gather before getting into the car…]

The suspect in example (8) from the United States data never actually admits to gang membership, which is an important element in this criminal case. Gang membership is treated differently by different states in the US, and some make it a criminal offense. This case is from the state of California, which makes it a crime to actively participate in a criminal street gang and to assist the gang in any felony and criminal conduct, and the conviction carries a potentially substantial financial and jail time punishment under the California Gang Affiliation Enhancement Law^9^. Gang membership also adds time to a sentence for a crime committed while a member, which was the case with this suspect who was accused of grievous bodily harm (GBH). The last speaking turn illustrates the assumption for the admission. The police officer asks the suspect where the group gathered before getting into the car, which indicates that gang group membership has been inferred as confirmed. We can see that later during the same interrogation the police officer makes multiple references to the gang and clearly indicates that he considers the suspect to be a member of that gang (e.g., by saying “tu y otros Sureños” = “you and other Sureño gang members”).

The same kind of apparent admission is observed in example (9) from our United Kingdom data, whereby the interviewing officer was apparently satisfied with an admission of guilt in relation to threatening communication (in a domestic abuse case) which was only implied (and which can easily be denied):

(9)
PO: Did you threaten to kill her?
S: She threatened to get her brother to kill me.
PO: Did her brother approach you that night?

As in the United States example in (8), in (9) the United Kingdom police officer never returns to the topic to confirm the apparent (*implied*) admission by the suspect to the accusation (of having threatened the female in question). We can make a strong assumption that the police officer took the suspect’s response above as an admission (by inference) because later in the conversation he asks questions that indicate this (e.g., “Did she threaten you first?”). It may be the case that there was further evidence in both cases (8) and (9) that would implicate both of these suspects in the alleged offenses (of criminal activity with gang membership and threatening communication respectively) so that the respective police officers did not need to elicit explicit confirmations and thus did not need to push for them. Nevertheless, eliciting the actual explicit admission would have added support for the prosecution, especially if further evidence was not available. Then again, even if further evidence was not available, there may have been a reason why the police officers in these two cases did not insist further on eliciting an explicit admission of guilt. Constant challenges, pressures and confrontations in a communicative exchange may result in failure to establish the all-important rapport and in the suspects shutting down and refusing to talk. Research has shown that preserving the communication channel is of crucial importance for evidence elicitation in policing and thus excessive insistence on explicit admissions may be counter-productive, because not losing your interlocutor altogether can still lead to more beneficial outcomes for the investigation even if a full confession or admission is not achieved (Musolff, 2019). The devil lies in the detail: when to press for admission and when to “let one slide,” as it were, which is something we comment on further in the “Conclusion and Limitations” section.

In the last two cases [examples (10) and (11)], both from the United States, we have inadvertent admissions of guilt that were a product of miscommunication due to inadequate translation:

(10)
PO1:. Did he, did he put his penis in that other gentleman’s mouth?
PO2: Okay, tu en cualquier tiempo pusistes tu cosa en la, en la boca del otro hombre?
[Okay, did you, did you at any time put your thing in the, in the other guy’s mouth?]
S: El quiso. Nadie lo ha forzado, él nos ofreció dinero porque quería hacer eso
[He wanted. Nobody forced him, he offered us money because he wanted to do that.]
PO2: He says nobody forced him, so…
PO1: I’m not asking him that. Did you put your penis in that fellow’s mouth?
PO2: So, él quiere saber si tú pusistes tu cosa en la boca de él.
[So, he wants to know if you put your thing in his mouth.]
S:… pues, él quiso, no, no, no la puse yo.
[… well, he wanted to, I didn’t, didn’t, didn’t put it there.]
PO2: Yes, because he wanted to.
PO1: Right. What happened after that?

(11)
PO1: Tú pushastes a la muchacha?^10^
[Did you push the young lady?]
S: Sí, le empujé las manos.
[Yes, I pushed her hands.]
PO1: La pushastes?
[Did you push her?]
S: Sí.
[Yes.]
PO1: Yes, he did.
PO2: Ok.

We can see that the police officers who acted as interpreters “aided” the admissions of guilt that were then accepted by the English monolingual police officers as given. In (10) the suspect denies his agency while the translation by the police officer in the last line confirms it and the other police officer understands it as such and moves the conversation to another topic. Similarly, in (11) the suspect denies that he pushed the girl – he only pushed *her hands* but the police officer insists on getting the *yes* or *no* answer in relation to any kind of pushing and he gets a “yes.” And *yes* as the answer to “did you push her?” is the only thing that the other police officer present gets because he was monolingual English-speaking and could not follow the exchange in Spanish. The bilingual police officer did not translate everything into English and thus an admission of guilt that the PO2 inferred as given was, in effect, a mistaken conclusion resulting from inadequate interpreting.

## Conclusion and Limitations

Our focus in this study has been on the specific types of confessions and admission of guilt that occur *inadvertently*, when suspects are not aware of what exactly they are confessing or admitting to, or when they are not explicitly confessing or admitting anything but appear to be doing so. Our data sampling was random, based on the selection (by the relevant law enforcement agencies) of cases to which access could be granted, and we discovered a relatively small number of confessions and admissions overall (17). Significantly, however, the *majority* of the confessions and admissions we identified were *inadvertent* (11 out of 17). This is a surprising finding because we did not know hitherto whether the phenomenon of inadvertent confessions (or admissions) was even present in real life situations such as these, let alone this frequent within a random sample. Our exploratory investigation was therefore risky, but ultimately highly successful – it has enabled us to detect and define this novel type of confession/admission as well as to identify and exemplify three general factors that are conducive to inadvertent confessions and admissions of guilt, which are: (i) general psycholinguistic processes of deriving meaning via *inferencing* (examples 5, 6, 7, 8, 9), (ii) *linguistic and cultural barriers* that, if not overcome, can result in unresolved or exploited misunderstandings (examples 2, 3, 4, 10 and 11), and (iii) *procedural features*, such as an adversarial questioning method, lack of legal representation and absence of professional interpreting (all United States cases: 1, 2, 3, 4, 8, 10, 11).

We have shown that inadvertent confessions and admissions occur in both jurisdictions, the United States and the United Kingdom, and that ZEP/LEP speakers are more likely to incriminate themselves inadvertently, without being aware of doing so, than monolingual speakers of the majority language, in this case English. Inadvertent confessions and admissions seem to occur more frequently in United States police interrogations than in United Kingdom police interviews, and it appears that lack of legal representation (after improper Mirandization; see Filipović, 2021 for further details) and an adversarial questioning method have a role to play here. However, we need to add caution to this statement because we need to bear in mind, again, the relatively small number of relevant cases analyzed in the current study. Furthermore, as one reviewer pointed out, the more serious criminal offenses in our United States data portion could also have contributed to the higher number of cases there. It is possible that certain types of crimes are more likely to result in aggressive questioning and in drawing inferences that are damaging for the suspect in both jurisdictions, and consequently lead to more inadvertent confessions or admissions of guilt. Our current dataset does not enable us to make any informed assumptions in this regard but it is certainly an avenue worth exploring in future research. Overall, in the light of the recent and urgent call to review and improve police interrogation practices in the United States (and also Canada; see Snook et al., 2021), we can see the present study as a contribution to the concerns that have been voiced about adversarial custodial interrogations.

It is important to emphasize that the miscommunication phenomena studied here take place in both monolingual and multilingual contexts, but that there appears to be more risk of them occurring if the communication takes place via an interpreter. Better quality interpreting is the answer for this problem, though even in cases when professional interpreting is available we still have issues stemming from the lack of perfect cross-linguistic, cross-cultural and cross-legal equivalents, which could lead to inadvertent admissions (or denials) of guilt (see also Filipović, 2007, 2019, 2021). This is why it is important to engage in adequate pre-interview briefing for all participants, as argued in the evidence-based training advice for United Kingdom police proposed in Mayfield (2017) and Filipović (2021). Explaining and defining the specific terms of the offense (which may subsume different descriptors even if the terms used are considered linguistically equivalent in translation), as well as alerting all interview/interrogation participants to the key linguistic and cultural information that needs to be borne in mind, would be advisable [for concrete examples of when and how this can be done see again Mayfield (2017) and Filipović (2019, 2021)].

It is also important to highlight here that “checking understanding” emerges as one of the key actions that can result in better evidence elicitation and avoidance, and in the prevention and resolution of misunderstandings that can lead to inadvertent confessions and admissions of guilt. An example of good practice is available in the case of United Kingdom Police Caution, where checking the understanding and elicitation of explicit descriptions by suspects of what each of the three portions of the Caution means is found in every United Kingdom interview (which is in stark contrast with the United States cases discussed here). Furthermore, we know that even native speakers find both Miranda and Caution problematic to understand (Shuy, 1997; Cotterill, 2000; Gibbons, 2001; Pavlenko, 2017) and that in interpreter-mediated police questioning situations this problem is compounded (Russell, 2001; Berk-Seligson, 2016; Filipović, 2021). Checking understanding cannot happen frequently throughout the police questioning event, especially in interpreter-assisted cases, because this would be very time-consuming. It can, however, be advisable to check understanding explicitly at least at key points in the interview or interrogation in order to ensure that suspects are fully aware of what exactly they are confirming or denying. And since, as we explained in the “Theoretical Background” section, reliance on inferences is the backbone of any communicative situation (Grice, 1989) and the potential for multiple inferences is vast, the devil lies in the detail of precisely how, and how often, to perform the checking of understanding by asking for it explicitly. This skill can be honed through professional training that is informed by empirical evidence and based on authentic, real-life examples such as the ones discussed in this study. Crucially, apparent concurrence on crucial matters for the investigation should not just be accepted as admission but queried and further probed.

It is our hope that this and similar research projects will raise awareness about the need to detect and resolve the inevitable inferential conflicts in police communication as well as overcome cross-linguistic, cross-cultural and procedural obstacles in access to justice, which will lead to elicitation of better-quality evidence and to improved safe-guarding against all unacceptable confessions, false, fabricated, coerced and inadvertent.

## Data Availability Statement

The datasets presented in this article are not readily available because the data access was granted solely to the author upon performance of strict security checks by the United Kingdom and United States authorities and the subsequent successful clearance confirmation. Requests to access the datasets should be directed to https://www.ndcalfpd.org and https://www.apccs.police.uk.

## Ethics Statement

The studies involving human participants were reviewed and approved by the GREC University of East Anglia. Written informed consent for participation was not required for this study in accordance with the national legislation and the institutional requirements.

## Author Contributions

The author is solely responsible for the fieldwork data collection and data analysis.

## Conflict of Interest

The author declares that the research was conducted in the absence of any commercial or financial relationships that could be construed as a potential conflict of interest.

## Publisher’s Note

All claims expressed in this article are solely those of the authors and do not necessarily represent those of their affiliated organizations, or those of the publisher, the editors and the reviewers. Any product that may be evaluated in this article, or claim that may be made by its manufacturer, is not guaranteed or endorsed by the publisher.

## Footnotes

1 The Reid Technique employed in United States policing comprises methods of questioning that focus on eliciting confession and include accusatory strategies as well as presentation of false evidence (see e.g., Milne and Bull, 1999 for more details and discussion).

2 PEACE is the acronym for Planning and preparation; Engage and explain; Account clarification and challenge; Closure; and Evaluation. It is not obligatory to follow it to the letter, but it is generally adopted as the best interview format in United Kingdom policing.

3 This study was done pre-PEACE and things have improved since then in United Kingdom policing, as Clarke and Milne (2001) demonstrate, though further improvements were apparently still needed.

4 The author of this study is the only person who had security clearance for access to all the data portions granted by the different United States and United Kingdom police authorities over a decade of data collection. Other researchers and collaborators of the author only had access to portions of the data depending on when they joined the research programme led by the author (*www.tacit.org.uk*). Thus, only a limited data portion was available for double-coding. This, however, is not an issue at present since there is no possible contention over any classification of items into different groups with multiple possible group memberships for individual items, where double-coding of the whole dataset would have been needed. At present we were just interested in detecting whether a confession or admission is there or not and how it came about, which was quite straightforward and thus the reliability rating is at 100%.

5 PO1-police officer/main investigator; PO2 – police officer/interpreter; S- suspect.

6 PO-police officer; S- suspect.

7 INT-Interpreter (in this case, a female family member).

8 LR-legal representative.

9 For exact wording of the relevant legal codes, definitions and case examples see: https://www.shouselaw.com/ca/defense/penal-code/186-22/.

10 PO1-police officer 1/interpreter; S- Suspect; PO2-police officer 2/main investigator.
